# Freeze-Induced Phase Transition and Local Pressure in a Phospholipid/Water System: Novel Insights Were Obtained from a Time/Temperature Resolved Synchrotron X-ray Diffraction Study

**DOI:** 10.1021/acs.molpharmaceut.3c00657

**Published:** 2023-10-27

**Authors:** Miguel A. Rodrigues, Olga Matsarskaia, Pedro Rego, Vitor Geraldes, Lauren E. Connor, Iain D. H. Oswald, Michael Sztucki, Evgenyi Shalaev

**Affiliations:** † Centro de Química Estrutural, Instituto Superior Tecnico, University of Lisbon, Lisbon 1049-001, Portugal; ‡ Institut Laue−Langevin, 71 Avenue des Martyrs, Grenoble 38000, France; § Strathclyde Institute of Pharmacy and Biomedical Sciences, University of Strathclyde, Glasgow G4 0RE, U.K.; ∥ Collaborative International Research Programme, University of Strathclyde and Nanyang Technological University, Singapore, Technology Innovation Centre, Glasgow G1 1RD, U.K.; ⊥ European Synchrotron Radiation Facility, Grenoble Cedex 9 38043, France; # Abbvie Inc., 2525 Dupont Drive, Irvine, California 92612, United States

**Keywords:** freezing, phospholipids, freeze-induced pressure, ice, small-angle X-ray scattering, synchrotron X-ray

## Abstract

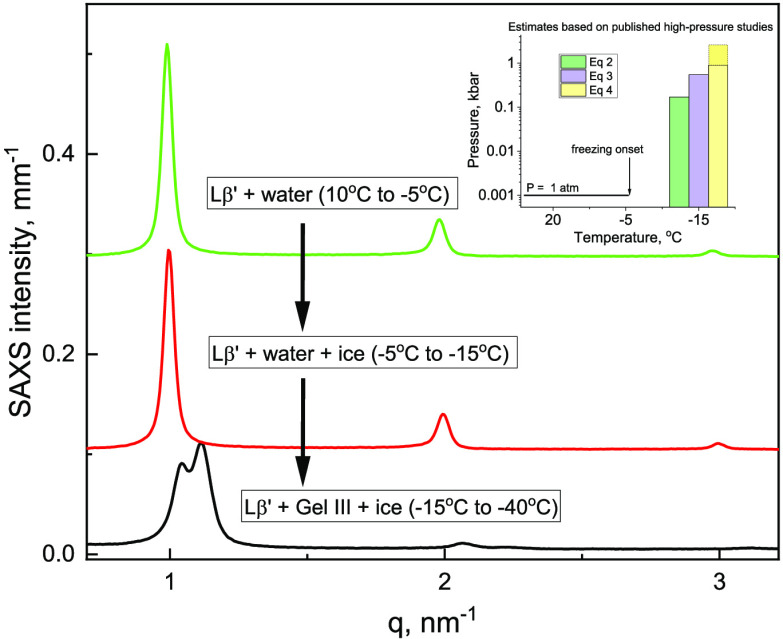

Water-to-ice transformation results in a 10% increase in volume, which can have a significant impact on biopharmaceuticals during freeze–thaw cycles due to the mechanical stresses imparted by the growing ice crystals. Whether these stresses would contribute to the destabilization of biopharmaceuticals depends on both the magnitude of the stress and sensitivity of a particular system to pressure and sheer stresses. To address the gap of the “magnitude” question, a phospholipid, 1,2-dipalmitoyl-*sn*-glycero-3-phosphocholine (DPPC), is evaluated as a probe to detect and quantify the freeze-induced pressure. DPPC can form several phases under elevated pressure, and therefore, the detection of a high-pressure DPPC phase during freezing would be indicative of a freeze-induced pressure increase. In this study, the phase behavior of DPPC/water suspensions, which also contain the ice nucleation agent silver iodide, is monitored by synchrotron small/wide-angle X-ray scattering during the freeze–thaw transition. Cooling the suspensions leads to heterogeneous ice nucleation at approximately −7 °C, followed by a phase transition of DPPC between −11 and −40 °C. In this temperature range, the initial gel phase of DPPC, Lβ′, gradually converts to a second phase, tentatively identified as a high-pressure Gel III phase. The Lβ′-to-Gel III phase transition continues during an isothermal hold at −40 °C; a second (homogeneous) ice nucleation event of water confined in the interlamellar space is detected by differential scanning calorimetry (DSC) at the same temperature. The extent of the phase transition depends on the DPPC concentration, with a lower DPPC concentration (and therefore a higher ice fraction), resulting in a higher degree of Lβ′-to-Gel III conversion. By comparing the data from this study with the literature data on the pressure/temperature Lβ′/Gel III phase boundary and the lamellar lattice constant of the Lβ′ phase, the freeze-induced pressure is estimated to be approximately 0.2–2.6 kbar. The study introduces DPPC as a probe to detect a pressure increase during freezing, therefore addressing the gap between a theoretical possibility of protein destabilization by freeze-induced pressure and the current lack of methods to detect freeze-induced pressure. In addition, the observation of a freeze-induced phase transition in a phospholipid can improve the mechanistic understanding of factors that could disrupt the structure of lipid-based biopharmaceuticals, such as liposomes and mRNA vaccines, during freezing and thawing.

## Introduction

Freezing is commonly used to improve the stability and extend the shelf life of various biopharmaceuticals. Most protein drugs are stored in the frozen state as drug substances.^1^ Freeze storage has also been used for commercial biotherapeutic products, for example, cell and gene therapy products including Glybera, IMLYGIC, and Luxturna, and mRNA anti-COVID 19 vaccines. While degradation rates are usually greatly decreased in the frozen state, freezing can nevertheless have a destabilizing effect on proteins, vaccines, and drug delivery systems such as liposomes and other lipid-based dosage forms.^2−4^ Several factors are commonly invoked to explain freeze-induced degradation of proteins, including freeze concentration, pH changes, and ice/solution interfaces.^5^ For lipid-based systems and biological membranes, destabilization mechanisms also include liquid crystalline-to-gel and lamellar-to-nonlamellar phase transitions and demixing of the membrane components.^6^ An additional mechanism of freeze-induced destabilization of biopharmaceuticals (biologicals) has been suggested more recently, that is, a freeze-induced increase in local pressure,^5^ based on two well-known facts. First, hexagonal ice has a higher specific volume than water, and therefore, freezing could lead to a significant increase in local pressure as the result of an increase in volume during water-to-ice transformation. The key point here is that the pressure increase depends on physical restrictions on the growth of ice crystals and on the flow of the remaining unfrozen amorphous phase, which is replaced and pushed away by growing ice crystals. Physical restriction to the expansion can be imposed by both the wall of the container and neighboring ice crystals, as well as via an increase in the viscosity of the freeze-concentrated solution. The second fact is that exposure of protein molecules to elevated pressure may lead to destabilization of a higher-order structure. Many proteins undergo unfolding and loss of activity at moderate hydrostatic pressures of less than 2 kbar.^7^ For example, a loss of almost 50% of the catalytic activity of carboxypeptidase Y was reported at a hydrostatic pressure of 0.5 kbar at 5 °C.^8^ A modest hydrostatic pressure of tens of bars could also lead to local conformational changes in protein molecules.^9^ In order to develop a comprehensive understanding of mechanisms of destabilization of biologicals during freeze–thaw, it would thus be essential to develop tools for quantifying the pressure increase during freezing.

Estimates of the freeze-induced pressure range from a few MPa (tens bar) to several kbar.^5^ A significant freeze-induced pressure buildup exceeding 2 kbar (2000 atm) can be achieved by sealing the solution in a container, such as a metallic capillary, and initiating freezing from one side of the container. This technique has been used to vitrify samples for electron microscopy^10−12^ and to supercool protein solutions for accelerated stability prediction.^13^ When ice crystals serve as the only physical constraint to expansion, as in water droplets frozen from outside, the pressure inside such droplets could also reach up to 2 kbar.^14^ A promising way to monitor freeze-induced pressure on a microscopic scale was recently suggested by Baccile et al. by using a lamellar probe, GC18:0.^18^ GC18:0 is a glycolipid with a β-d-glucose headgroup linked to the C17 carbon of stearic acid via a glycosidic bond.^19^ In the Baccile et al. 2020 study,^18^ the lamellar space period (*d*-spacing) was measured by small-angle X-ray scattering (SAXS) under controlled freezing conditions. The *d*-spacing was correlated with osmotic pressure based on separate isothermal osmotic stress measurements performed by the water vapor gas phase equilibration at different water activities at 25 °C. The osmotic pressures were estimated to be approx. 1 at −15 °C and 3.5 kbar at −60 °C. It was also demonstrated that the *d*-spacing (and the corresponding osmotic stress) during freezing depends on the distance of the lipid probe from the ice crystals; the closer the probe is to the ice crystals, the higher the osmotic stress.^18^ In a conventional freezing setup, when the sample can macroscopically expand in at least one dimension (usually upward) during freezing, microscopic freeze-induced pressure was estimated by monitoring shifts in Bragg diffraction peaks of ice, resulting in pressure estimates between 2 and 3.5 kbar.^15−17^ However, much lower values of freeze-induced pressure, between 10 and 70 bar (0.01 and 0.07 kbar), were reported in other studies of conventionally frozen systems where strain gauges were used.^5,20^ This major discrepancy of 2 orders of magnitude difference in freeze-induced pressure for pharmaceutically relevant freezing conditions represents a significant gap in the understanding of freeze-induced pressure as a potential mechanism of destabilization of proteins and other biological systems during freeze–thaw and freeze-drying.

A traditional way to directly measure hydrostatic pressure is to use a substance with a known pressure dependence of spectroscopic or structural properties. Such a high-pressure probe is added to a particular system, and the spectral/structural properties of the probe are monitored while the pressure is applied. Ruby crystals (aluminum oxide doped with Cr^3+^) are a standard pressure probe where fluorescence has linear response changes as a function of hydrostatic pressure.^21^ Unfortunately, while ruby is a very consistent pressure marker above 1 GPa (10 kbar), the accuracy at pressures below 1 GPa is lower.^22^ The lack of experimental probes to measure pressure in the range of interest for freeze-induced pressure phenomena (i.e., 0.1–3 kbar) is a second gap in the characterization of the freezing process.

We suggest that a common synthetic phospholipid, 1,2-dipalmitoyl-*sn*-glycero-3-phosphocholine (DPPC), can serve as a probe to detect and quantify an increase in pressure during freezing. DPPC is one of the most studied lipid systems in which multiple phases can be formed as a function of temperature and pressure. At ambient pressure and above 0 °C in the fully hydrated state (i.e., at water concentrations above 30 wt %), the following DPPC phases are typically observed: liquid crystalline lamellar Lα, lamellar gel Lβ′, gel with wave-like ripples Pβ′, and lamellar crystalline Lc phases.^23,24^ Additional information on the phase relationships in the DPPC/water system can be found in the Supporting Information. Furthermore, relationships have been established between the hydrostatic pressure and DPPC structure. At elevated pressures of up to 2 kbar, a lamellar interdigitated phase, LβI, was observed at 25 °C,^25^ while three additional high-pressure gel phases (Gel III, Gel IV, and Gel V) were detected in a pressure range of 1.7–12.5 kbar and temperatures between 10 to 60 °C.^26^ In addition, application of hydrostatic pressure imposes gradual changes in structural dimensions of the Lβ′ phase of DPPC, with a linear relationship observed between pressure and interlamellar spacing.^26^

In the present study, the phase behavior of the DPPC/water system is studied by small- and wide-angle X-ray scattering (SAXS/WAXS). Silver iodine is used as an ice nucleating agent to attenuate the stochastic nucleation behavior associated with water supercooling. We observed that freezing resulted in a phase transition from the Lβ′ phase to a low-temperature DPPC phase, tentatively identified as the Gel III phase. The Gel III phase appears between −11 and −15 °C, approximately 5 °C below the heterogeneous ice nucleation. The Lβ′-to-Gel III conversion continues during cooling and is further enhanced by homogeneous ice nucleation in the aqueous interlamellar space of the Lβ′ phase. The homogeneous ice nucleation is detected at approximately −40 °C by DSC. Considering the high-pressure nature of the Gel III phase, we propose that DPPC has the potential to serve as a probe to detect and quantify freeze-induced pressure. Furthermore, the observation of the freeze-induced phase transition in a phospholipid could also be relevant for understanding the impact of freezing on lipid nanoparticles and lipid drug delivery systems, considering that many pharmaceutical and biopharmaceutical formulations contain phospholipids (e.g., cytarabine liposome injection and lipid nanoparticle delivery systems). Several such products have been recently approved by the regulatory agencies in the USA and in the EU.^27^

## Experimental Section

1,2-Dipalmitoyl-*sn*-glycero-3-phosphocholine (98% purity) was purchased from TCI (Belgium) as a powder. The suspensions were prepared in deionized water by adding an appropriate mass of DPPC (final concentrations are 5 or 10 wt %) using 0.5 mL Eppendorf tubes, and 10 freeze–thaw cycles were performed in three steps: immersing the vials in liquid nitrogen for 10 s, thawing in a water bath at 50 °C for 60 s with gentle agitation, followed by homogenization in a vortex (20 s at 50 W). For each concentration, four independent samples were prepared. SAXS peak intensities for three of the eight DPPC/water samples were low (patterns not shown), and they were not used in the present study.

Suspensions were loaded into 1.0 mm quartz capillary tubes (WJM, Germany) with a few particles of silver iodine placed on the bottom of the capillary to act as an ice nucleating agent, attenuating the stochastic nucleation behavior associated with water supercooling. The particles were placed on the bottom of the capillary, far from the illuminated section, but still in contact with the liquid that was loaded afterward with a syringe. The fill volume was less than 20% of the capillary volume, with a sufficient place for the sample to expand upward during freezing.

SAXS–WAXS experiments were performed on the ID02 beamline at the European Synchrotron Radiation Facility (ESRF) in Grenoble, France, at a radiation wavelength λ of 0.995 Å.^28^ Two-dimensional SAXS and WAXS images were simultaneously recorded using a Rayonix MX-170HS and Rayonix LX-170HS CCD detector at sample-to-detector distances of 1 m and 12.2 cm, respectively. The exposure time was adjusted to use the dynamic range of the detector (<0.1 s). The CCD images were corrected for detector artifacts and normalized to absolute units using standard procedures.^28^ The filled capillaries were loaded onto a Linkam temperature-controlled stage THMS600/TMS94. The SAXS-WAXS patterns were recorded every 2 s for cooling and heating rates of 5 °C/min in the temperature range between 10 and −30 °C or −40 °C.

The analysis of SAXS and WAXS data was performed using the batch peak analysis function in Origin 2018 software. In addition, several patterns were analyzed individually to check the quality of the fitting results. The peaks were fitted to a Voigt function to determine peak maxima and peak areas. The lamellar repeat spacing, *d*, is calculated from the SAXS data as
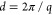
1where *q* is the position of maxima of the first SAXS peak.

The peak areas are used to estimate fractions of the corresponding phases. Note that the use of the X-ray diffraction peak areas for phase composition would provide a semiquantitative estimation because diffraction intensity depends on several factors, including mass absorption coefficients, densities of the phases, and preferred orientation of the crystals, in addition to the mass fractions of the phases.^29^ Density depends on both phase structure and temperature; the Lα-to-Pβ′ transition of DPPC, for example, results in approximately 4% decrease in specific volume, while the Pβ′-to-Lβ′ transformation leads to a further decrease in the specific volume, although of a magnitude of less than 1%.^30^ The temperature dependence of the specific volume is also noticeable, with a decrease of the specific volume of approximately 1% over a 15 °C range.^31^

Differential scanning calorimetry (DSC) experiments were performed using a NETZSCH DSC 200 F3Maia. The DSC instrument was calibrated using bismuth, tin, and indium at a scanning rate of 10 °C/min. The 10 wt % DPPC/water samples for the DSC study were prepared in the same way as samples for the SAXS/WAXS experiment. Approx. 10 mg of the DPPC suspension, either with or without addition of AgI, was loaded in hermetically sealed aluminum pans, and DSC scans were carried out by cooling from 20 to −60 °C, followed by heating at scanning rates 1, 5, and 10 °C/min.

## Results

**Figure 1:**
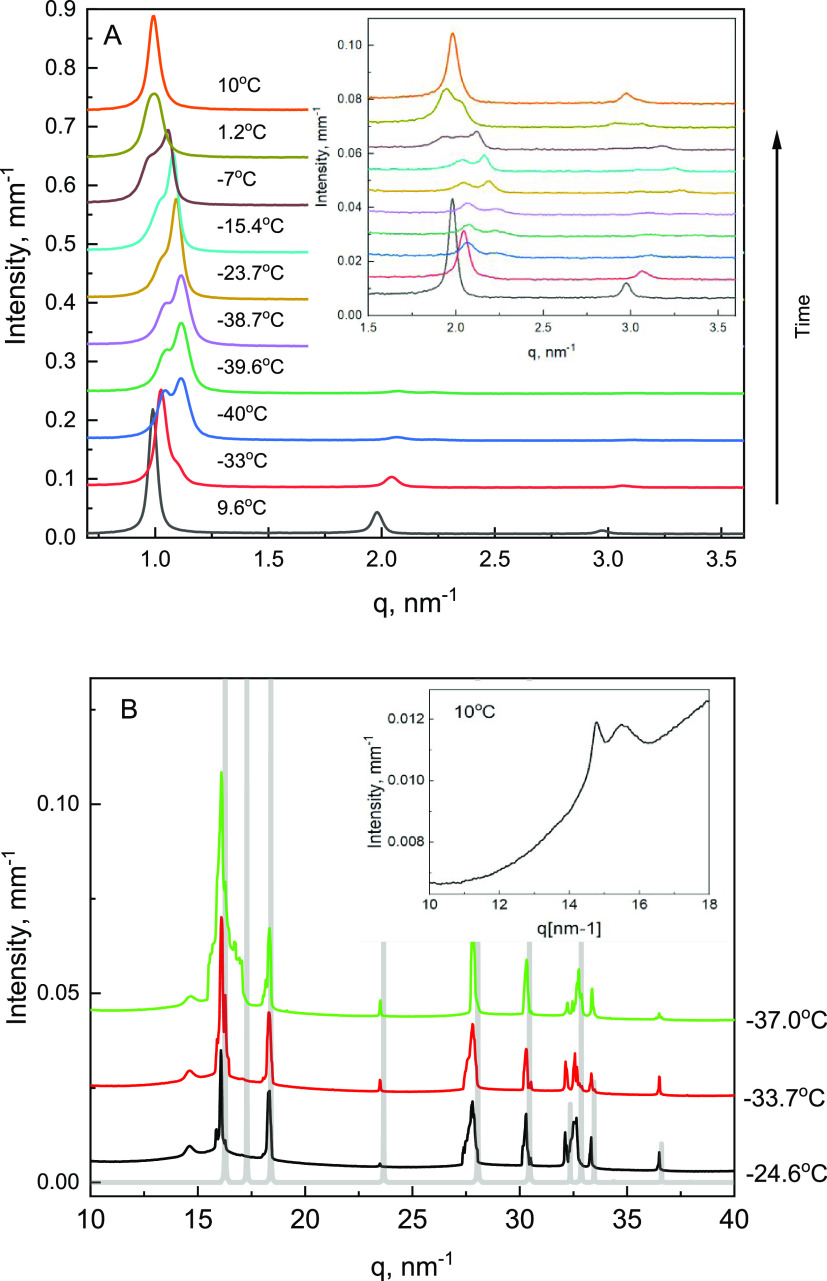
Representative SAXS (A) and WAXS (B) patterns collected during cooling of 10 wt % DPPC. The reference Ih pattern, which is generated from the reported structure,^36^ is also shown in (B) as semitransparent gray lines. Insets show a magnified portion of the SAXS patterns to illustrate changes in the 2nd and 3rd lamellar peaks (A), and part of the WAXS pattern at 10 °C to show two peaks of the Lβ′ phase (B). The SAXS and WAXS curves are shifted vertically, with the exception of the first curves on the bottom of both figures. The absolute intensity scale on the *y*-axis corresponds to the initial pattern in each graph.

A combination of SAXS and WAXS data is used for the detection and identification of phase transitions during cooling/heating of DPPC/water suspensions. In the 10% DPPC sample, three peaks are observed in the initial SAXS pattern in the *q* range from 0.7 to 3.6 nm^–1^(Figure 1A), with peak positions at *q* ratios of 1:2:3 indicative of the lamellar structure.^32^ This SAXS pattern is consistent with the Lβ′ (gel) phase, as expected.^33^ The initial (before freezing) WAXS pattern confirms this phase assignment; two DPPC-related reflections, which are observed at 14.8 nm^–1^ (*d*_20_) and 15.5 nm^–1^ (*d*_11_), are indicative of the Lβ′ phase (Figure 1B, inset).^33,34^ On cooling the suspension from 9.6 to −4.5 °C, there are gradual changes to the WAXS peak positions; specifically, the *d*_20_ and *d*_11_ reflections move from 14.8 to 14.65 nm^–1^ and from 15.5 to 15.7 nm^–1^, respectively. The *d*_20_ reflection contracts while the *d*_11_ reflection (Figure 1B, inset) expands over the same cooling period (Figure S3A,B in the Supporting Information). The same trend in the WAXS pattern during cooling was reported in the literature.^35^ SAXS patterns remain similar in this temperature region. Overall, while there are minor changes in structural dimensions and orientations of DPPC molecules, no phase changes are observed during cooling from 9.6 to −4.5 °C, with the DPPC remaining in the Lβ′ phase.

On cooling below −5.5 °C, a characteristic peak of hexagonal ice, Ih, is detected in the WAXS patterns, indicative of an ice nucleation event. Upon further cooling, additional WAXS peaks appear; the peaks in the WAXS patterns of the frozen samples are aligned with the theoretical pattern of hexagonal ice, Ih (Figure 1B, semitransparent gray lines). One additional weak peak, which is observed in several WAXS patterns (Figure S3C in the Supporting Information), could belong to the nucleation agent AgI. In the SAXS region, a second set of peaks is observed in the frozen samples (Figure 1A, pattern at −40 °C), which is indicative of the DPPC phase transition. This low-temperature phase, labeled Gel III (the phase identification is discussed below), is also lamellar, as characterized by reflections with position ratios of 1:2. The Gel III phase appears initially as a higher-q shoulder on the first peak of the Lβ′ phase in the SAXS pattern, which grows into a separate peak during the isothermal hold at −40 °C (Figure S1 in the Supporting Information). The Gel III phase coexists with the Lβ′ phase during heating and converts back to the Lβ′ phase after thawing between 1.2 and 10 °C (Figure 1A and Table S1). Similar results are obtained in two separate SAXS experiments performed with independently prepared 10 wt % DPPC suspensions; specifically, the Gel III phase forms at −12 to −15 °C. Again, this phase coexists with the Lβ′ phase during subsequent cooling and heating (Figure S2 in the Supporting Information).

**Figure 2:**
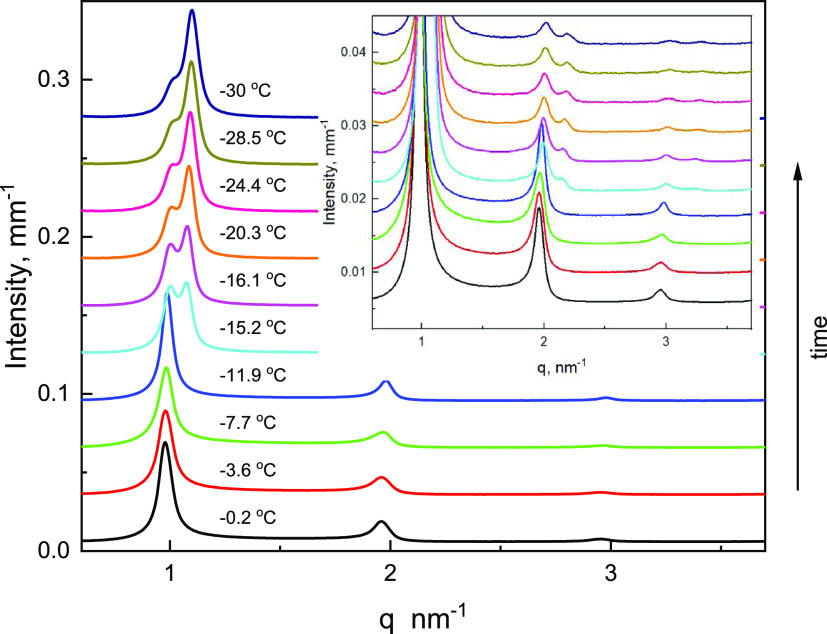
Representative SAXS patterns during cooling of a 5 wt % DPPC suspension. Magnified SAXS patterns are shown in the inset to illustrate the evolution of the 2nd and 3rd peaks. The curves are shifted vertically, with the exception of the first curve on the bottom. The absolute intensity scale on the *y*-axis corresponds to the initial pattern in each graph.

The transition from Lβ′ to the Gel III phase during cooling is detected by SAXS in the 5 wt % DPPC samples as well (Figures 2 and S4 in the Supporting Information). The Gel III phase appears between −13.6 and −14.4 °C, first as a shoulder of the main peak (the SAXS pattern is not shown) and then as a separate peak at −15.2 °C (Figure 2). The second and third characteristic peaks of the lamellar structure at the *q* values of 1:2 and 1:3 appear at −15.2 and −16.1 °C, respectively (Figure 2).

**Figure 3:**
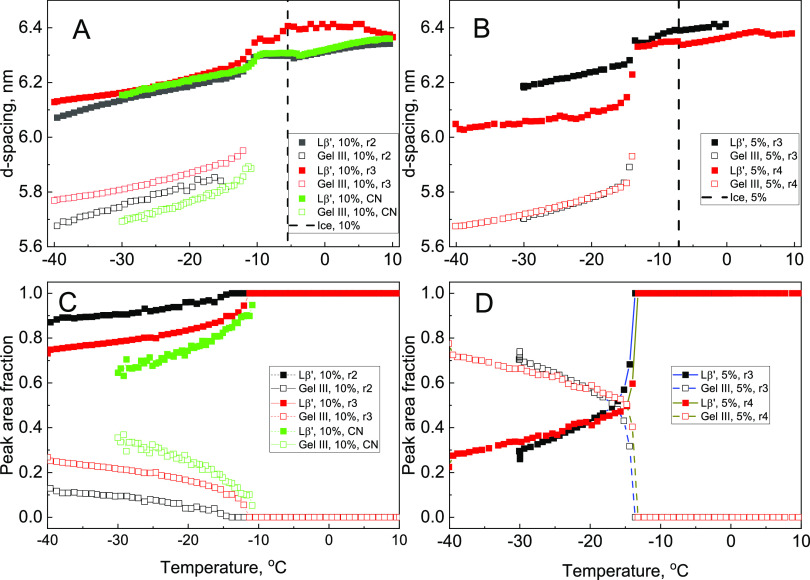
Lamellar lattice constant, which corresponds to the first reflection and is calculated as *d* = 2π/*q*, (A, B) and change in the Lβ′ and Gel III fractions (C, D) during cooling of the DPPC/water suspensions with 10 wt % (A, C), and 5 wt % (B, D) DPPC. The phase fractions are expressed using the areas of the 1st SAXS peak for corresponding phases. The filled squares are for Lβ′, and the open squares are for the Gel III phase. Different colors represent individual sample preparations and SAXS/WAXS temperature runs, with labels r2, r3, r4, and CN used for tracking purposes. The vertical lines in (A, B) mark the ice nucleation temperature in 10 and 5 wt % suspensions, respectively.

Changes in the lamellar repeat spacing during cooling are summarized in Figure 3A,B. While there is variability in the *d*-spacing values between repeat runs, temperature trends are qualitatively consistent between the samples studied. During initial cooling, the lamellar repeat distance remains approximately constant with the *d*-spacing between 6.3 and 6.4 nm in both 10 and 5 wt % DPPC samples (Figure 3A,B, respectively). Note that the ice nucleation event (marked as vertical lines in Figure 3A,3B) does not trigger any immediate change in the temperature trends of the interlamellar spacing. The second phase, Gel III, is formed between −11 and −15 °C (Figure 3A,3B, the appearance of the second set of curves with *d* < 6 nm). The temperature gap between ice nucleation (vertical lines in Figure 3A,3B at −6 to −7 °C) and the appearance of the Gel III phase (−11 to −15 °C) may be reflective of the growth of ice crystals after initial nucleation, causing a buildup of local pressure and transformation to the Gel III phase.

The gradual decrease in the *d*-spacing is observed for both phases during cooling from approximately −15 to −40 °C. While the specific physical mechanism of these changes has not been established, it is probably related to the pressure buildup, although the role of freeze-induced dehydration^37^ cannot be ruled out. Note that the impact of dehydration on the *d*-spacing is not straightforward, with dehydration having two opposite effects on the *d*-spacing of the Lβ′ phase. It would reduce the thickness of the interlamellar water layer^37,38^ while also increasing the tilting angle of the hydrocarbon chains at the same time; the increased tilting angle would increase the *d*-spacing, while the thinner water layer would decrease it. As a result of these two competing effects of dehydration, the *d*-spacing of the Lβ′ phase of DPPC was found to be essentially constant (*d* = 64 Å) between 30 wt % and 70 wt % water,^30^ while it decreases to 59 Å at 20 wt %.^39^ We also note that while a simple temperature-dependent contraction might also be a possibility, we consider this unlikely because of a very weak temperature dependence of *d*-spacing in the absence of water phase transition, with the *d*-spacing of the gel phase reported to be essentially independent of temperature.^30,40^

The transition to the Gel III phase occurs in both 5 and 10 wt % DPPC/water suspensions, while the Lβ′ phase coexists with the Gel III phase in each case. The difference lies in the percentage conversion to the Gel III phase. For each sample, we have been able to extract the peak areas for β′ and the Gel III phase as an indication of the conversion (Figure 3C,3D). While the peak areas do not indicate the exact phase composition of the samples (see the Experimental Section), they can be used to compare the extent of the phase transition between different samples. We have observed that the Lβ′-to-Gel III phase transition is much sharper in the 5 wt % DPPC sample, which has higher water content (95 wt % water vs 90 wt % water in the 10 wt % DPPC suspension), with the Gel III peak area fraction exceeding 0.4 over a narrow temperature interval (less than 2 °C cf. 10 °C for 10 wt %). In the 5 wt % DPPC samples, the fraction of Gel III approaches 0.7 while remaining below 0.4 in the 10 wt % DPPC samples. The difference in the speed and extent of the phase conversion probably reflects the quantity of ice formed in each of the suspensions, which is obviously larger in the suspension with the lower (5 wt %) DPPC concentration. The larger quantity of ice crystals nucleating and growing will be reducing the locally accessible volume around the β′ phase, causing the transformation to the Gel III phase. Therefore, the observation of faster and more complete phase transformation in the samples with the higher ice amount (i.e., in the 5 wt % samples) is consistent with the freeze-induced pressure hypothesis.

**Figure 4:**
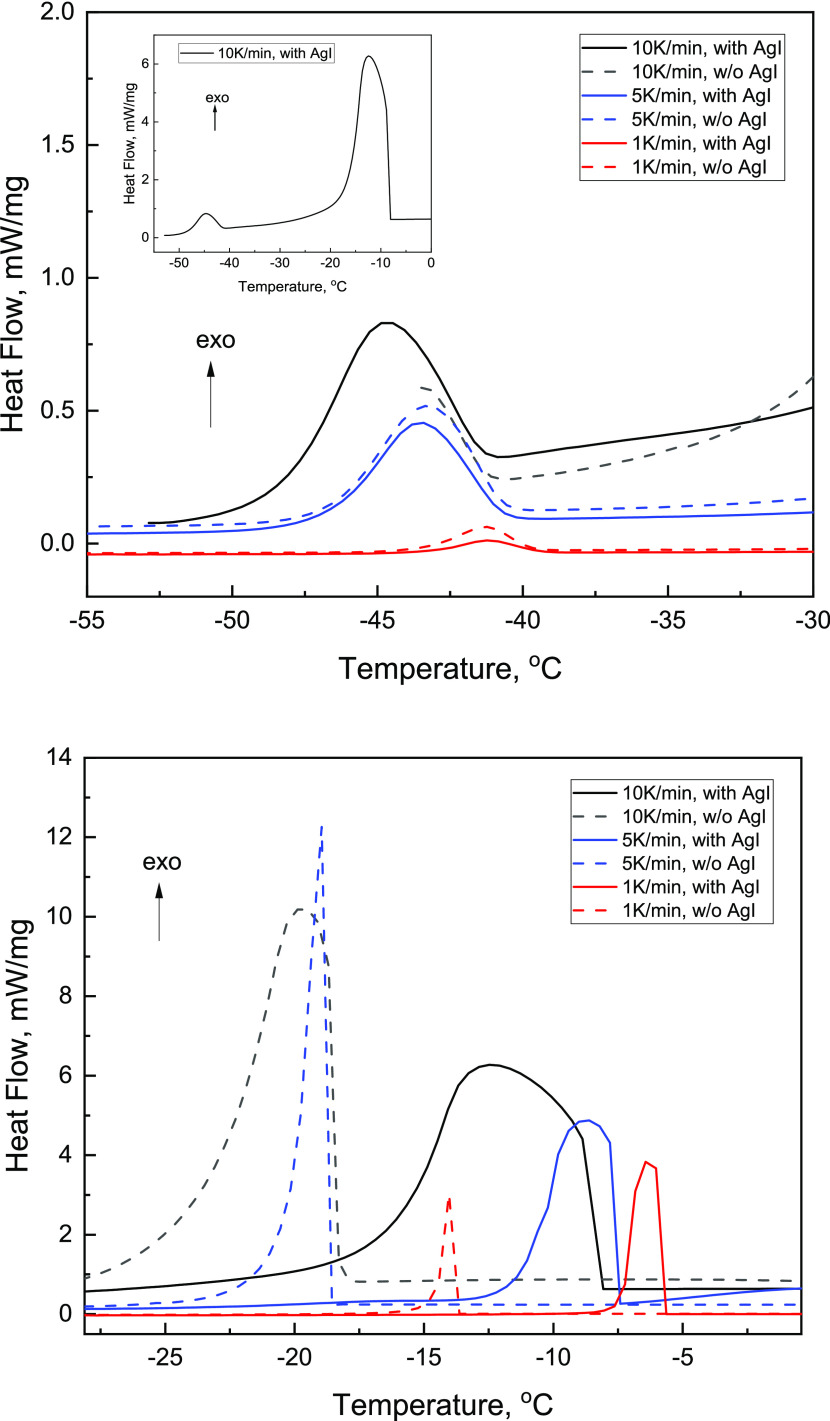
DSC cooling scans for 10 wt % DPPC/water mixtures without AgI and with AgI at scanning rates of 1, 5, and 10 °C/min. An example of an entire DSC curve is shown in the inset (top). The bottom and top graphs represent heterogeneous and homogeneous ice nucleation events, respectively.

In addition to the SAXS and WAXS studies, we performed DSC experiments with 10% DPPC samples (Figure 4). During cooling, two exothermic events are observed. The first event reflects heterogeneous ice nucleation, while the second (weaker) exotherm at approximately −40 °C is due to homogeneous ice nucleation of water in the interlamellar space.^41,42^ No thermal events related to the DPPC phase transition are observed during freezing, probably because the freeze-induced DPPC transition is spread over a wide temperature range. The addition of the ice nucleation agent, AgI, leads to a major decrease in the supercooling (i.e., an increase in the heterogeneous ice nucleation temperature; Table S2 in the Supporting Information), while neither the presence of the ice nucleation agent nor cooling rates influence the homogeneous ice nucleation temperature.

## Discussion

Freezing of the DPPC/water suspensions leads to the formation of a second lamellar phase, which is identified as the Gel III phase. The phase assignment is based on the similarity between the freeze-induced SAXS/WAXS changes in the present study and those reported in ref (26) for pressure-induced transformation of Lβ′ to the Gel III phase. Specifically, the reduction in the lamellar *d*-spacing (average 0.46 ± 0.05 nm, Figure 3) is similar to the literature-reported SAXS change for the pressure-induced Lβ′-to-Gel III transition in which *d*-spacing decreases by approximately 0.4 nm.^26^ In the WAXS results of this study, the *hk* 20 WAXS peak of DPPC (*d*_20_, *q* = 14.8 nm^–1^) is not impacted by the freeze-induced formation of the Gel III phase. The temperature dependence of the position of the *hk* 20 peak is flat after the Gel III phase has been formed (Figure S3B in the Supporting Information). Similarly, no changes in the *d*_20_ diffraction were reported during the Lβ′-to-Gel III pressure-induced phase transition.^26^ Note that the *hk* 11 WAXS diffraction of the Gel III phase (*d*_11_, *q* = 15.5 nm^–1^), which could provide additional support to identify the Gel III phase,^26^ overlaps with strong Ih peaks, and hence, it cannot be used for more comprehensive phase comparison. Overall, both SAXS and WAXS results for the freeze-induced DPPC phase transition, as observed in this study, are consistent with the pressure-induced Lβ′-to-Gel III transition, as reported in the literature. Two alternative interpretations of the freeze-induced SAXS/WAXS changes are addressed in the Supporting Information, with the conclusion that they are not consistent with the experimental observations.

A significant feature of the freeze-induced DPPC phase transition is that the transition is incomplete, with the newly formed lamellar Gel III phase coexisting with the original Lβ′ phase. There is also a temperature–time gap between ice nucleation and the DPPC phase transition. While the temperature trends in the SAXS data are found to be reproducible and consistent between samples studied, there is a noticeable difference in both *d*-spacing values and the extent of the phase transition, as described in Figure 3. This variability would indeed be expected if the DPPC phase transition is caused by growing ice crystals. The freeze-induced local pressure would depend on the specifics of ice crystal growth (e.g., speed and shape of the water crystallization front), which are probably different between samples. While ice nucleation is promoted by using an ice nucleation agent, AgI, the direction and rate of progression of the crystallization front are not controlled in this study.

It is also worth noting that the DPPC phase transition, as detected by SAXS, continues during the isothermal hold at −40 °C (Figure S1). In DSC experiments, an exothermic event is detected at approximately −40 °C (Figure 4); this transition is related to crystallization of water in the interlamellar space.^41,42^ This second freezing event would result in additional volume expansion and therefore additional pressure buildup. Taken together, these two observations, i.e., the DSC-detected freezing of interlamellar water and the increase in the fraction of the Gel III phase at approximately −40 °C observed by SAXS, are consistent with the hypothesis of the freeze-induced phase transition of DPPC, in which the driving force is suggested to be local pressure due to the volume expansion as the result of water-to-ice transformation, as discussed above.

As a semiquantitative estimate of the freeze-induced pressure using the SAXS data of this investigation, two independent approaches are used here. One way is to use the literature-reported pressure dependence of the Lβ′-to-Gel III phase transition. The pressure dependence of the Lβ′/Gel III transition in DPPC was proposed to be linear with *dT/dP* = 34.6 °C/kbar between −30 and 30 °C based on a Raman spectroscopy study.^43^ A similar linear *T*/*P* relationship for the Lβ′/Gel III phase boundary was reported based on the experimental data above 10 °C with synchrotron SAXS and FTIR (the Lβ′ = Gel II).^26^ The corresponding equations for the Lβ′/Gel III *T*/*P* phase boundary can be written as follows
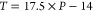
2
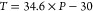
3where *T* is the temperature in °C and *P* is the pressure in kbar.

Equation 2 is approximated from the graphical *T*/*P* phase diagram reported in ref (26), and eq 3 is from the data reported in ref (43). Taking into account the onset temperature of Lβ′ to Gel III in this study (−11 °C), the freeze-induced local pressure corresponds to either 0.17 kbar (eq 2) or 0.55 kbar (eq 3).

An alternative way to estimate freeze-induced pressure is to use the freeze-induced SAXS *d*-spacings for the Lβ′ phase (Figure 3). The lamellar repeat spacing of the Lβ′ phase is sensitive to hydrostatic pressure, with the pressure dependence of the lamellar lattice constant, *a*, described as^26^
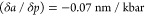
4

The experimentally observed decrease in the lamellar *d*-spacing of the Lβ′ phase during freezing is approximately 0.06 ± 0.01 nm^–1^ (average of four samples), while one of the 5 wt % DPPC suspensions has a larger (0.18 nm^–1^) decrease in *d*-spacing (Figure 3 and Table S1 in the Supporting Information). From eq 4, the freeze-induced pressure corresponds to approximately 0.9 kbar for a *d*-spacing decrease of 0.06 nm^–1^ and 2.6 kbar for a 0.18 nm^–1^ decrease.

## Conclusions

In this study, the freeze-induced phase transition from the Lβ′ phase to a low-temperature phase, tentatively identified as the Gel III phase, is observed in DPPC/water suspensions. The Gel III phase was previously reported to form under elevated hydrostatic pressure,^26,43^ and therefore, its formation indicates an increase in local pressure during freezing. During our experiments, the freeze-induced Lβ′-to-Gel III transition is incomplete, which could indicate sample heterogeneity with the implication that the localized pressure is different between different parts of the sample. The results therefore indicate that freeze-induced pressure represents a nonequilibrium situation, resulting from the combination of volume expansion during the water-to-ice transformation and the physical constraints to the expansion, which are imposed by neighboring ice crystals and the container walls.

We propose, furthermore, that DPPC could be a suitable molecular probe to detect the local pressure during the freezing of aqueous systems, while a knowledge gap needs to be addressed for such applications. Specifically, there is an uncertainty in the location of the Lβ′/Gel III phase boundary in the *T*/*P* phase diagram of the DPPC/water system, with a significant difference between two literature sources.^26,43^ In addition, while our results are consistent with an observation of the Gel III phase, there might be other lower-temperature/higher-pressure phases in the DPPC/water system (e.g., Driscol et al.^44^ reported another high-pressure DPPC phase, Gel X, in a high-pressure NMR study of 15% *d*_62_-DPPC dispersion in water). These aspects can be addressed in a separate SAXS/WAXS study of DPPC/water mixtures under elevated hydrostatic pressure at temperatures below 0 °C to confirm if the Gel III phase is the only low-temperature/high-pressure phase forming during freezing and to establish the pressure/temperature phase boundary for the Lβ′/Gel III equilibria at subzero temperatures. Orthogonal techniques, such as NMR or Raman spectroscopy, may provide further assistance in the characterization of the phase composition of the frozen DPPC/water system. Finally, considering that the kinetics of the Lβ′-to-Gel III phase transition could depend on specific patterns of ice nucleation and ice crystal growth, it would be important to monitor nucleation and progression of the ice front in investigations of freeze-induced phospholipid phase transitions.

To summarize, the novel findings of this study are as follows: (i) the Lβ′ phase of a phospholipid (DPPC) is converted during freezing to another lamellar gel phase, tentatively identified as the high-pressure Gel III phase; (ii) while the Lβ′-to-Gel III transition is caused by freezing, there is a temperature/time lag between the ice nucleation event and the DPPC phase transition; (iii) the phase transition is incomplete, and the two DPPC phases coexist at temperatures below −15 °C; (iv) the driving force of the Lβ′-to-Gel III transformation is proposed to be an increase in the local hydrostatic pressure caused by the volume expansion during water-to-ice conversion; and (v) the freeze-induced local pressure range is estimated to be 0.2–2.6 kbar. Overall, the results of this study support the hypothesis of freeze-induced pressure as an additional mechanism of freeze-induced destabilization of proteins, membranes, and lipid-based drug delivery systems to complement widely recognized effects of freeze concentration and ice/solution interfaces.

## Disclosures

E.S. is an employee of AbbVie. He participated in the design of the study, in the analysis and interpretation of data, and in writing, reviewing, and approval of the publication. AbbVie contributed to the design of the study, data analysis and interpretation, and manuscript preparation; AbbVie did not provide financial support for the study. M.A.R., V.T., and V.G. are employees of the University of Lisbon, O.M. is an employee of the Institut Laue–Langevin, L.E.C. is an employee of Novartis, I.D.H.O. is an employee of the University of Strathclyde, and M.S. is an employee of European Synchrotron Radiation Facility. They do not have any conflicts of interest.

## Supporting Information Available

Figures: SAXS patterns of the 10 wt % DPPC sample during isothermal hold at −40 °C; additional SAXS patterns of 10 and 5 wt % DPPC suspensions during cooling and heating; and WAXS patterns of the 10 wt % DPPC sample during cooling and their overlays with characteristic peaks of the orthorhombic crystalline phase of DPPC. Tables: Change in the *d*-spacing of the first SAXS peak of the Lβ′ phase upon the Lβ′-to-Gel III phase transition during cooling; temperature region of the Gel III-to-Lβ′ transition during warming; and temperatures of ice nucleation events during cooling of the 10 wt % DPPC/water mixture by DSC. Text: summary on polymorphism of DPPC; alternative interpretations of SAXS changes during cooling; and a brief discussion of freeze-induced pressure (PDF)
