# Supplementary material for: Freeze-Induced Phase Transition and Local Pressure in a Phospholipid/Water System: Novel Insights Were Obtained from a Time/Temperature Resolved Synchrotron X-ray Diffraction Study
Source: Mol Pharm. 2023 Oct 27;20(11):5790–9. doi: 10.1021/acs.molpharmaceut.3c00657 (PMC10630958; doi:10.1021/acs.molpharmaceut.3c00657)
Supplement: Supplementary file 1 — mp3c00657_si_001.pdf [file mp3c00657_si_001.pdf]

Supporting Information

Freeze-induced phase transition and local pressure in a phospholipid/water system: Novel insights from time/temperature resolved synchrotron X-ray diffraction study.

Miguel A. Rodrigues,[1] Olga Matsarskaia,[2] Pedro Rego,[1] Vitor Geraldes,[1] Lauren E. Connor,[3,4] Iain D. H. Oswald,[3]* Michael Sztucki,[5] Evgenyi Shalaev[6]*

[1]Centro de Química Estrutural, Instituto Superior Tecnico, University of Lisbon, Lisbon, Portugal.
[2] Institut Laue–Langevin, 71 Avenue des Martyrs, 38000 Grenoble, France.
[3] Strathclyde Institute of Pharmacy and Biomedical Sciences, University of Strathclyde, Glasgow G4 0RE, U.K.
[4] Collaborative International Research Programme, University of Strathclyde and Nanyang Technological University, Singapore, Technology Innovation Centre, Glasgow G1 1RD, U.K.
[5] European Synchrotron Radiation Facility, 38043 Grenoble, Cedex 9, France.
[6] Abbvie Inc., Irvine, California 92612, United States.

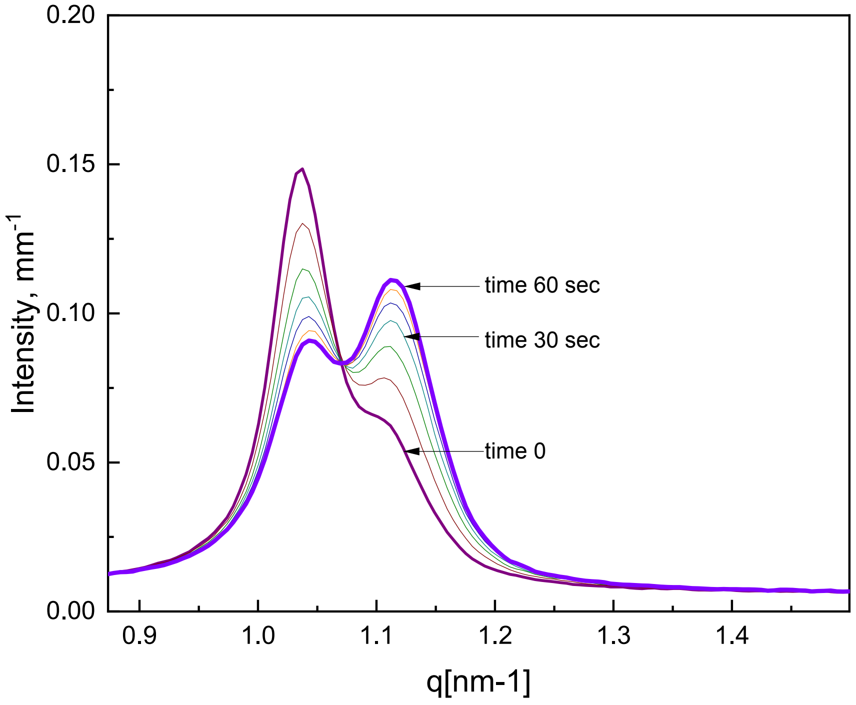

Figure S1. SAXS curves of DPPC 10 wt% sample during isothermal hold at -40°C. The patterns are collected every 10 sec.

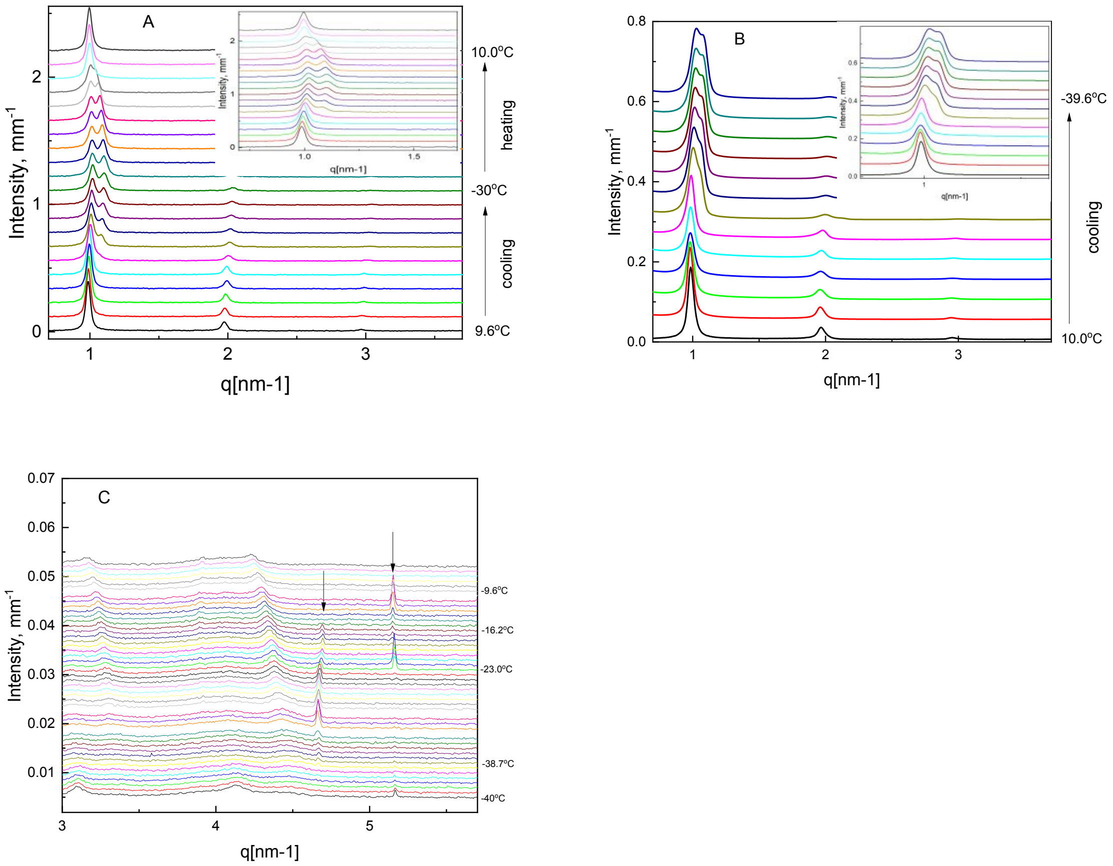

Figure S2. SAXS patterns of independently prepared DPPC 10 wt% suspensions. The curves are shifted vertically. (A): sample labelled “controlled nucleation”; (B): sample labelled as run-3. (A,B): The insets show the evolution of the first reflection. (C): Higher-q region of SAXS patterns of one of the DPPC 10 wt% sample during heating. Two weak SAXS peaks, which do not belong to either the Lβ’ or to the Gel III structure, are detected at q 4.67 and 5.23 nm^{-1} during heating from -38.7 to -16.2°C and -23.0 to -9.6°C, respectively. These peak are not observed in the two other 10 wt% DPPC samples tested in this study. The SAXS peaks may indicate the appearance of an additional minor phase; the extra peaks are sharp and narrow, which would indicate a crystalline nature of the phase. To check this hypothesis, X-ray scattering patterns for three additional DPPC phases (phases 1, 2, and 3 as per Albon’s nomenclature) are calculated using the unit cells reported in ref [S1]. One of these phases, crystalline phase 2, has peaks at locations similar to those of the two unidentified peaks in the experimental patterns at q = 4.67 and 5.23 nm^{-1}. However, Albon’s phase 2 is lamellar phase, and it has a strong first peak at approx. 1.1 nm^{-1}. If the unknown peaks belong to the Albon’s phase-2, there should be a strong peak at ~1.1 nm^{-1}, in addition to 2 peaks of the Lβ’ and Gel III phases. There is no evidence of the third peak (Figure 1, main manuscript file), therefore, Albon’s phase-2 is unlikely to form in this sample. Note also that Albon’s phase-2 is stable in the temperature range of this study, it undergoes a phase transition above 63°C.

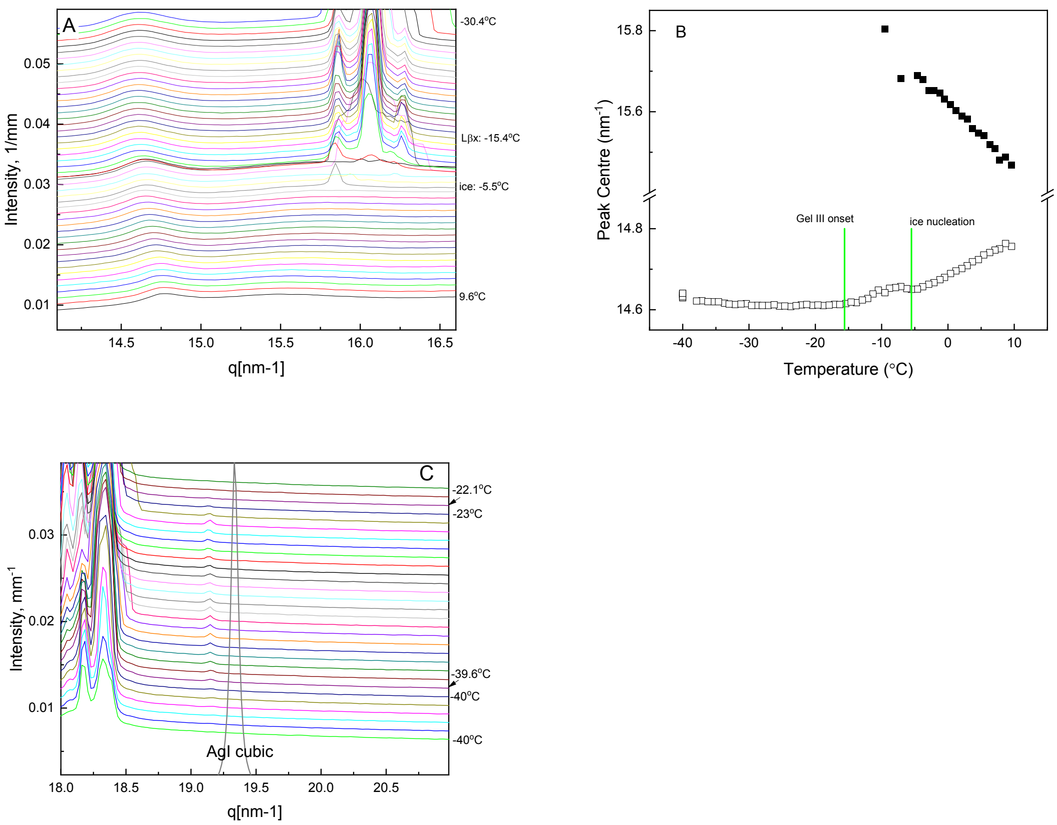

Figure S3. (A) WAXS patterns of the 10 wt% DPPC suspension during cooling. The curves are shifted vertically. (B) d-spacing of two WAXS peaks of the DPPC gel phase during cooling of the DPPC 10 wt% sample. (Hollow squares: sharp peak; filled squares: broad shoulder). The broad shoulder could not be fit after the nucleation of ice due to the peak position coinciding with ice peak positions. (C) magnified portions of the WAXS patterns of the 10% DPPC suspension during heating showing a minor unidentified peak; the peak position is similar to one of the peaks of AgI. The position in the sample capillary and temperature difference of the reference sample may alter the observed position of the AgI peak. In some WAXS patterns, very broad and strong scattering is detected in the q range of approx. 15 to 20 nm^{-1}; this is probably due to the strong scattering from ice crystals, causing detector saturation.

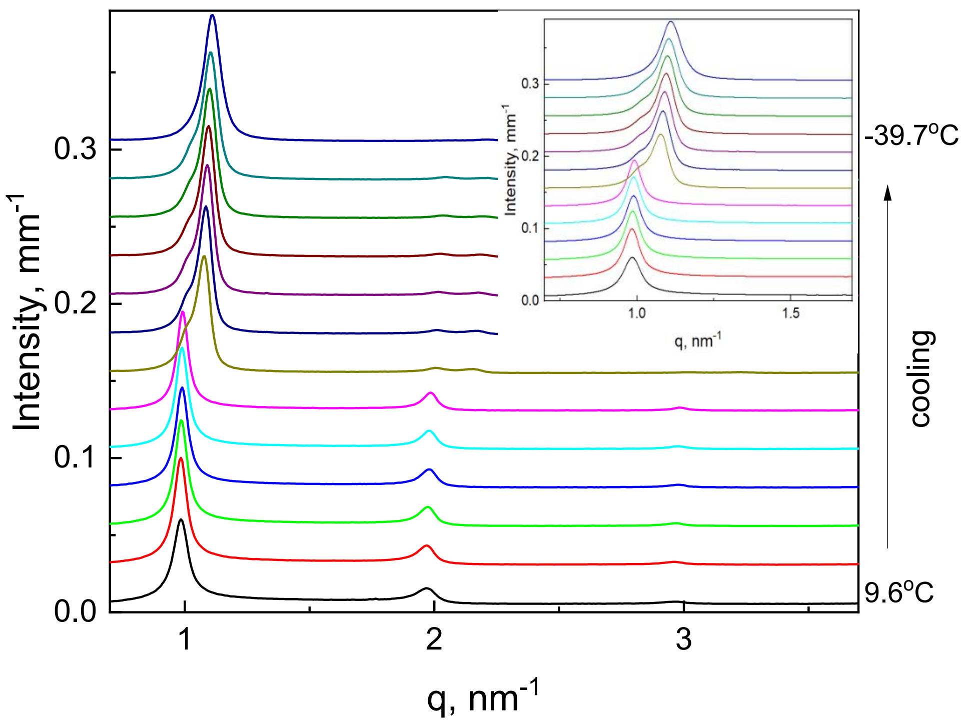

Figure S4. SAXS patterns of the second sample of the DPPC 5 wt% suspension during cooling. The inset show evolution of the first reflection. The curves are shifted vertically.

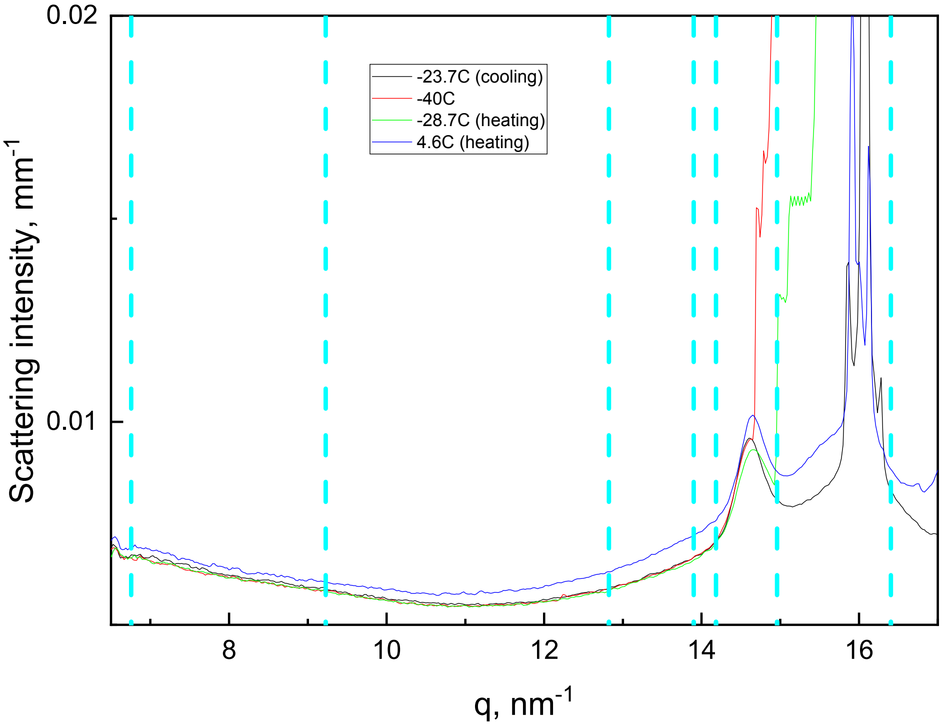

Figure S5. Examples of experimental WAXS patterns for 10 wt% DPPC (solid lines) and characteristic peaks of the orthorhombic crystalline phase of DPPC[S2] (vertical dashed lines).

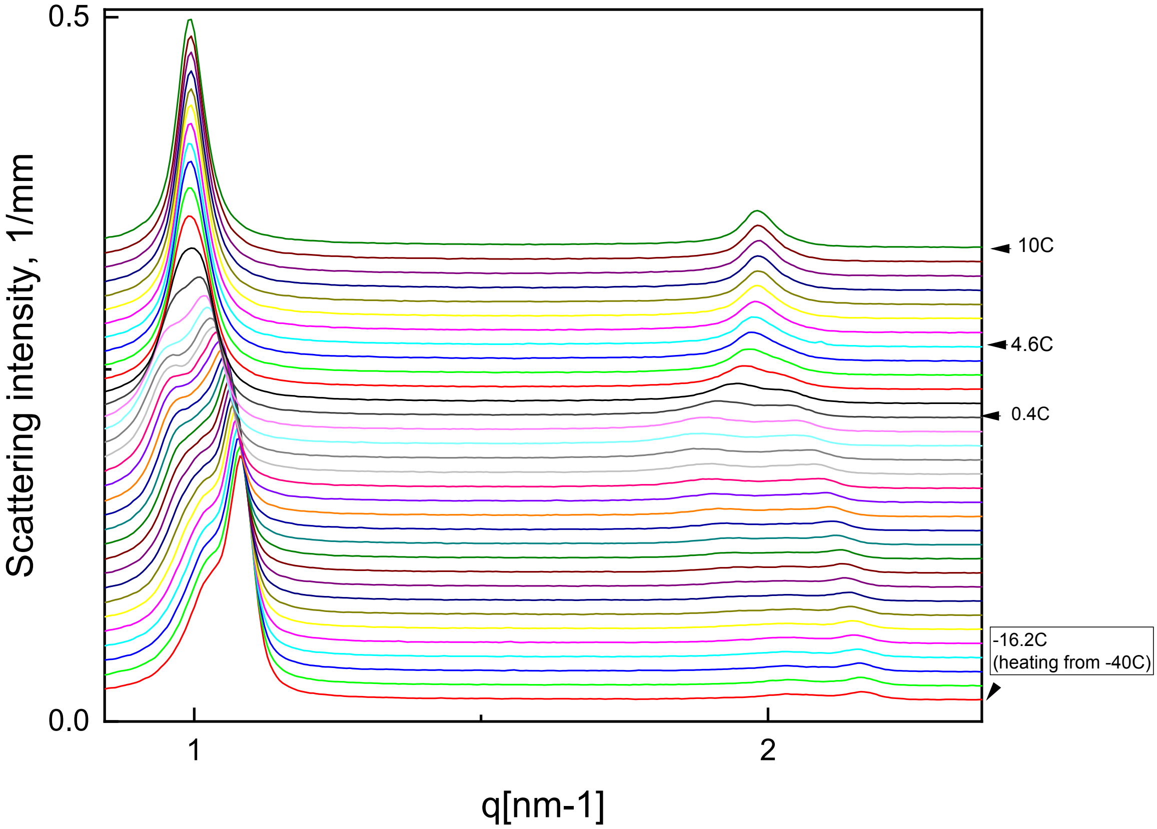

Figure S6. SAXS patterns of 10 wt% DPPC suspension showing Gel III to Lβ’ transition upon heating.

Table S1. Change in the d-spacing of the first SAXS peak of the Lβ’ phase upon the Lβ’ to Gel III phase transition during cooling, and the temperature region of the Gel III to Lβ’ transition during warming.

Sample label	Change in the Lβ’ peak position during cooling, nm^{-1}	temperature of the Gel III to Lβ’ transition during warming
10%, CN	0.05	-1.2 to 0.4°C
10%, run 2	0.05	1.2 to 4.6°C
10%, run 3	0.06	-5.4 to 4.6°C
5%, run 3	0.06	Not tested during warming
5%, run 4	0.18	Not tested during warming

Table S2. Temperatures of water crystallization events during cooling of 10 wt% DPPC/water mixture by DSC. The results are consistent with the published studies, with heterogeneous ice nucleation without ice nucleating agent observed between -10°C and -20°C[S12-S14] , and a second exothermic thermal event at approx. -40°C to -50°C.[S9, S12, S15]

Sample	Cooling Rate, °C/min	Tice-1 onset (heterogeneous), °C	Tice-2 onset (homogeneous), °C
with AgI	1	-6.2, -6.2*	-39.9, -41.2*
	5	-7.4	-40.7
	10	-8.3	-41.3
without AgI	1	-13.9	-39.7
	5	-18.6	-40.7
	10	-18.2	-40.6

*two independent samples

Additional comments.

Polymorphism of DPPC.

Phospholipids demonstrate a diverse polymorphism. In this respect, they are unique among other organic molecules. Even simple binary phospholipid-water systems form multiple phases. For example, more than 15 phases were reported for DPPC, 1,2-dipalmitoyl-sn-glycero-3-phosphocholine (DPPC). Specifically, the following DPPC phases are typically observed above 0°C at ambient pressure in the fully hydrated state (i.e., at water concentrations above 30 wt%): liquid crystalline lamellar Lα, lamellar gel Lβ', gel with wave-like ripples Pβ', and lamellar crystalline Lc phases,[S16, S17] The Lc phase was reported to be an undecahydrate containing 11 molecules of water per DPPC molecule, DPPC*11H_2O,[S3] while the gel phase was proposed to have 5 or 6 water molecules closely interacting with the polar headgroups.[S18, S19] Furthermore, up to twelve DPPC phases have been identified.[S1] Phase composition in phospholipids in general and DPPC/water system in particular depends on the composition (i.e., water content for binary systems) and temperature, and is described using temperature-composition (T-x) phase diagrams. Several T-x phase diagrams for the DPPC-water system have been published, which reflect phase relationships above 0°C and cover water content range from zero to 100 wt%.[S1, S16, S17, S20]

The structure of the Gel III phase, based on the Raman spectroscopy, is characterized by monoclinic interchain packing, and the Lβ' to Gel III transition (Gel II = Lβ') involves a damping of the inter- and intrachain rotational orientations.[S10] Furthermore, the Gel III phase was suggested to have a lower tilt angle, smaller head group area, and lower hydration than the Lβ' phase.[S11]

Rejected alternative interpretations of the SAXS changes during freezing of DPPC suspensions.

The SAXS patterns (Figures 1, 2) show the appearance of a second lamellar structure with a shorter interlamellar distance, the Gel III phase, in frozen DPPC/water samples below -15°C. From a general perspective, a shorter interlamellar distance for a phospholipid lamellar phase can be either due to (i) freeze-induced dehydration of the gel phase as water molecules diffuse from the lamellar phase to growing ice crystals,[S21] or (ii) a phase transition. To consider the former possibility (i.e., a dehydrated Lβ' structure), note that water molecules are distributed between two phases, bulk water phase and DPPC phase. Freezing (water-to-ice conversion) results in a partial dehydration of the Lβ' phase of DPPC, as a fraction of interlamellar water molecules migrate to the bulk ice phase.[S21] Such freeze-induced dehydration can have two opposite effects on the d-spacing of the Lβ' phase. The dehydration would reduce thickness of the interlamellar water layer,[S21] while also increasing the tilting angle of the hydrocarbon chains at the same time; the increased tilting angle would increase the d-spacing[S22], while the thinner water layer would decrease the d-spacing. As the result of these two competing effects of dehydration, the d-spacing of the Lβ' phase of DPPC was found to be essentially constant (d = 64 Å) between 30 wt% to 70 wt% water[S3], while it decreases to 59 Å at 20 wt%[S9], with these measurements performed above 0°C. However, the consistent co-existence of 2 lamellar structures, as observed in this study, would be difficult to explain in the "Gel III = dehydrated Lβ' phase" scenario. Therefore, the Gel III probably corresponds to a new lamellar phase, and not to a dehydrated Lβ' phase.

The sharp decrease in the lamellar d-spacing during freezing of DPPC/water suspensions was suggested by Kiselev et al to be due to formation of the Lc phase.[S13] Our results do not support the identification of the new phase as the Lc phase, as follows. The Lc phase is characterized by a 3-

dimensional order, with several characteristic WAXS diffraction peaks. [S2] Positions of the Lc WAXS peaks are compared with several representative WAXS patterns with the Gel III phase from this study in Figure S6 (Supporting information). No Lc-specific peaks are observed in the experimental WAXS patterns indicating its absence. It should be noted that possible preferred orientation effect may result in a reduced diffraction intensity of some peaks, but the absence of the number of reflections does still constitute a strong case against the Lc phase. The final argument against the "Lc phase" interpretation comes from the comparison of the temperature/time conditions of the Lβ'/Lc phase transition from the published studies.[S2, S3,S8, S9, S23-S26] The Lc phase represents thermodynamically stable phase, in respect to the Lβ' phase, below 15°C. It means that, if the Lc phase forms during freezing, as proposed in [S13], the Lc would persist until heating to 15°C. Instead, we observed that the phase labelled as the Gel III phase in our study (called the Lc phase in Ref[S13]) converts to the Lβ' phase during heating around 0°C, which is well below the temperature of the Lc-to-Lβ' phase transition (15°C). Additional arguments against the "freeze-induced Lc phase" hypothesis are provided in the next paragraph.

The Lc phase in DPPC was observed to form during isothermal holds at temperatures -4°C to 13°C with annealing time ranging from minutes to days.[S2-S8] In DSC experiments, a characteristic thermal event, which can be considered to be a "signature" of the Lc phase, was detected after a relatively short annealing of 40 min at -2°C. The enthalpy of the thermal event corresponding to the transition increased with the hold time and reached constant value after >40 hours.[S3] Ruocco & Shipley observed changes in the WAXS patterns over a similar time frame, with the main WAXS peak moved from 4.18 Å to ~ 4.25 Å after hold for >10 min at -2°C, followed by a more gradual increase to 4.40 Å.[28] The shoulder moved quickly from 4.08 Å to ~ 4.0 Å, and then more slowly to 3.87 Å. Conversely, no changes in the SAXS patterns were detected during the first 1.5 h.[S3] Collectively, the Lβ' to Lc transition was reported to be observed first by WAXS during relatively long (tens and hundreds of minutes) isothermal hold, while changes in the SAXS patterns took even longer to develop. In this study, the freeze-induced DPPC phase is detected by SAXS, while no major changes in the WAXS main peak (approx. 4.24 nm^{-1}) are observed at the same time (Figure S3, Supporting information), which is opposite to the sequence of WAXS/SAXS changes for the Lβ' to Lc phase transition described in the literature.[S3] The relatively fast kinetics of freezing-triggered DPPC phase transition is, with the timescale of minutes, also plays against the "Lc phase" interpretation, as a slow kinetics of formation has been considered to be a main signature of the Lc phase in both DPPC [S3] and other phospholipids [S27, S28] Arguably the most important point against the "freeze-induced Lc?" hypothesis is the temperature range of the existence of the Lc phase. The freeze-induced phase is formed during cooling to below -11°C (Figure 3, main manuscript), and converts back to the Lβ' phase around 0°C during heating (Table S1, Figure S6, Supporting Information), while the Lc phase would be expected to persist above 0°C. The Lc phase is stable up to 15°C and converts into the gel phase upon heating between 15 °C and 20°C.[S2] The gap in the stability temperature range provides a strong argument against the "freeze-induced Lc phase" interpretation. This conclusion is also consistent with an earlier assessment by Grunert etal[S9], who observed a formation of "the subzero temperature phase" (probably the same as the Gel III = freeze-induced phase of this study) with a lower d-spacing of 58.5 Å in DPPC samples and with a water content 23 wt% to 50 wt% at approx. -10°C to -15°C, and suggested that this phase is different from the phase associated with the "sub-transition" (which is the Lc phase).

Freeze-induced pressure. When ice crystals form, the liquid phase is displaced by growing ice crystals due to the volume expansion as the result of water-to-ice conversion. An increase in pressure is not expected if the unfrozen portion of the sample can expand and flow freely, i.e., when it forms a continuous liquid phase with no physical constrains for the expansion. Any constrains to the liquid flow and expansion would lead to a corresponding increase in the hydrostatic pressure, with the unfrozen liquid serving to transmit the pressure. Furthermore, if the unfrozen fraction of the sample converts to a glass, the "pressure" term is no longer applicable because there is no medium for pressure transmission. In this scenario, a more appropriate terminology would be internal material stress instead of pressure. To distinguish between these two cases, i.e., hydrostatic pressure and internal material stress, it would be essential to known the glass transition temperature, Tg, of the unfrozen fraction. In the DPPC/water system, the Tg is -25 to -40°C, depending on the water content of DPPC in equilibrium with ice.[S29] Therefore, for the freeze-induced DPPC phase transition, which starts at approx. -15°C (i.e., above the Tg), the unfrozen fraction can serve as the pressure transmission medium. In addition, there is unfrozen water in the interlamellar space, which can also transmit pressure. The situation would change at -40°C with the interlamellar water freezing, and the DPPC gel phase entering glassy state. Therefore, both liquid transmission media would cease to exist, and anisotropic internal material stress could develop in the DPPC phase.

References.

S1. Albon, N. Transitions and molecular packing in highly purified 1,2-dipalmitoyl-phosphatidylcholine–water phases. I. Transitions, improved phase diagrams, method of packing analysis, molecular structures of diglyceride, and polar regions. *J Chem Phys*. 1983, *78*, 4676.

S2. Ruocco, M. J.; Shipley, G. G. Characterization of the sub-transition of hydrated dipalmitoylphosphatidylcholine bilayers. X-ray diffraction study. *Biochim. Biophys. Acta*. 1982, *684*, 59-66.

S3. Ruocco, M. J.; Shipley, G. G. Characterization of the sub-transition of hydrated dipalmitoylphosphatidylcholine bilayers. Kinetic, hydration and structural study. *Biochim. Biophys. Acta*. 1982, *691*, 309-320.

S4. Fuldner, H. H. Characterization of a Third Phase Transition in Multilamellar Dipalmitoyllecithin Liposomes. *Biochemistry*. *1981*, 20, 5707-5710.

S5. Stumpel, J.; Eibl, H.; Nicksch, A. X-ray analysis and calorimetry on phosphatidylcholine model membranes. The influence of length and position of acyl chains upon structure and phase behavior. *Biochim. Biophys. Acta*. 1983, *727*, 246-254.

S6. Akiyama, M. X-Ray Diffraction Studies of Subtransition in Dipalmitoylphosphatidylcholine. *J. Appl. Phys. (Japan).* 1985, *24*, 231-234.

S7. Akiyama, M.; Matsushima, N.; Terayama, Y. Kinetics of the Subtransition of Multilamellar Dipalmitoylphosphatidylcholine*. J. Appl. Phys. (Japan).* 1987, *26*, 1587-1591.

S8. Tristram-Nagle, S.; Suter, R. M.; Sun, W-J.; Nagle, J. F. Kinetics of subgel formation in DPPC: X-ray diffraction proves nucleation-growth hypothesis. *Biochim. Biophys. Acta, Biomembranes*. 1994, *1191*, 14-20.

S9. Grunert, M.; Borngen, L.; Nimts, G. Structural phase transition due to a release of bound water in phospholipid bilayers at temperatures below 0° C. *Ber Bunsenges Phys. Chem*. 1984, *88*, 608-612. http://dx.doi.org/10.1002/bbpc.19840880707.
S10. Wong, P. T. T.; Siminovitch, D. J.; Mantsch, H. H. Structure and properties of model membranes: new knowledge from high-pressure vibrational spectroscopy. *Biochim. Biophys. Acta*. 1988;947:139-171. http://dx.doi.org/10.1016/0304-4157(88)90023-8.
S11. Czeslik, C.; Reis, O.; Winter, R.; Rapp, G. Effect of high pressure on the structure of dipalmitoylphosphatidylcholine bilayer membranes: a synchrotron-X-ray diffraction and FT-IR spectroscopy study using the diamond anvil technique. *Chem. Phys. Lipids*. 1998, *91*, 135–144. http://dx.doi.org/10.1016/S0009-3084(97)00104-7
S12. Kristiansen, J.; Hvidt, A. Lipid bilayers at subzero temperature studied by differential scanning calorimetry. *Cryo-Letters*. 1990, *11*, 137-142.
S13. Kiselev, M. A.; Lesieur, P.; Kiselev, A. M.; Ollivon, M. Ice formation in model biological membranes in the presence of cryoprotectors. *Nuclear Instruments and Methods in Physics Research A*. 2000, *448*, 255-260. http://dx.doi.org/10.1016/S0168-9002(99)00730-5
S14. Bronshtein, V. L.; Steponkus, P. L. Calorimetric studies of freeze-induced dehydration of phospholipids. *Biophys. J*. 1993, *65*, 1853-1865. http://dx.doi.org/10.1016/S0006-3495(93)81250-5
S15. Ladbrooke, B. D.; Chapman, D. Thermal analysis of lipids, proteins, and biological membranes. Review and summary of some recent studies. *Chem. Phys. Lipids*. 1969, 3, 304-367. http://dx.doi.org/10.1016/0009-3084(69)90040-1.
S16. Kodama, M. Phase transition phenomena induced by the successive appearances of new type of aggregation states of water molecules in L-dipalmitoylphosphatidylcholine-water system, *Thermochim. Acta*. 1986, *109*, 81-89. http://dx.doi.org/10.1016/0040-6031(86)85010-9.
S17. Matsuki, H.; Goto, M.; Tada, K.; Tamai, N. Thermotropic and Barotropic Phase Behavior of Phosphatidylcholine Bilayers. *Int. J. Mol. Sci*. 2013, *14*, 2282-2302; doi:10.3390/ijms14022282
S18. Gawrisch, K.; Arnold, K.; Gottwald, T.; Klose, G.; Volke, F. Deuterium NMR studies of the phosphate-water interaction in dipalmitoyl phosphatidylcholine-water systems. *Studia Biophys*. 1978, *74*, 13-14.
S19. Tricot, Y.; Niederberger, W. Water orientation and motion in phospholipid bilayers: a comparison between oxygen-17 and deuterium NMR. *Biophys. Chem*. 1979, *9*, 195-200. http://dx.doi.org/10.1016/0301-4622(79)85001-2.
S20. Grabielle-Madelmont C, Perron R. Calorimetric studies on phospholipid-water systems. I. DL-Dipalmitoylphosphatidylcholine (DPPC) – water system. *J Colloid Interface Sci.* 1983;95:471-482. https://doi.org/10.1016/0021-9797(83)90207-2.
S21. Gleeson, J. T.; Erramilli, S.; Gruner, S. M. Freezing and melting water in lamellar structures. *Biophys. J*. 1994, *67*, 706-712. http://dx.doi.org/10.1016/S0006-3495(94)80531-4.
S22. Tardieu, A.; Luzzati, V.; Reman, F. C. Structure and Polymorphism of the Hydrocarbon Chains of Lipids: A Study of Lecithin-Water Phases. *J. Mol. Biol*. 1973, *75*, 711-733. http://dx.doi.org/10.1016/0022-2836(73)90303-3
S23. Füldner, H. H. Characterization of a Third Phase Transition in Multilamellar Dipalmitoyllecithin Liposomes. *Biochemistry*. 1981, *20*, 5707-5710. http://dx.doi.org/10.1021/bi00523a011.
S24. Stümpel, J.; Eibl, H.; Nicksch, A. X-ray analysis and calorimetry on phosphatidylcholine model membranes. The influence of length and position of acyl chains upon structure and phase behavior. *Biochim. Biophys. Acta*. 1983, *727*, 246-254. http://dx.doi.org/10.1016/0005-2736(83)90410-8.

S25. Akiyama M. X-Ray Diffraction Studies of Subtransition in Dipalmitoylphosphatidylcholine. *J. Appl Phys (Japan).* 1985, *24*, 231-234. http://dx.doi.org/10.1143/JJAP.24.231.
S26. Akiyama, M.; Matsushima, N.; Terayama, Y. Kinetics of the Subtransition of Multilamellar Dipalmitoylphosphatidylcholine*. J. Appl. Phys (Japan).* 1987, *26*, 1587-1591. DOI 10.1143/JJAP.26.1587
S27. Shalaev EY, Zografi G, Steponkus PL. Occurrence of Glass Transitions in Long-Chain Phosphatidylcholine Mesophases. *J Phys Chem B.* 2010;114:3526-3533. http://dx.doi.org/10.1021/jp910348y
S28. Shalaev EY, Steponkus PL. Phase diagram of 1,2-dioleoylphosphatidylethanolamine (DOPE):water system at subzero temperatures and at low water contents, *Biochim Biophys Acta.* 1999;1419:229-247. http://dx.doi.org/10.1016/S0005-2736(99)00068-1.
S29. Shalaev, EY, G Zografi, PL Steponkus (2010) Occurrence of Glass Transitions in Long-Chain Phosphatidylcholine Mesophases. *J Phys Chem B* 114, 3526-3533.
